# Effect of practice on the control of reach extent

**DOI:** 10.1007/s00221-025-07181-x

**Published:** 2025-10-25

**Authors:** Faith N. Schroers, Troy M. Herter, Dylan Bruemmer, Takeo Ichiyanagi, Austin Hertherington, Michael O’Donnell, Janelle Ozorowski, Chad Simmons, Jill Campbell Stewart

**Affiliations:** Department of Exercise Science, Arnold School of Public Health, University of South Carolina, 921 Assembly Street, Room 301E, Columbia, SC 29208 USA

**Keywords:** Motor control, Reaching, Planning, Feedback, Dominance, Practice

## Abstract

The control of reaches to targets that vary in distance involves a combination of anticipatory planning and feedback-based adjustments. However, it is not known if their contributions to the control of reach extent change with repetitive practice. This study investigated the effect of three days of practice on the control of reach extent. Right-hand dominant participants reached with either the non-dominant left arm or dominant right arm to six targets presented in two directions and three distances in a virtual environment. The effect of practice on planning and feedback-based adjustments to control reach extent was examined by determining how well peak acceleration and time to peak velocity predicted the eventual distance moved, respectively. Both arm groups demonstrated improvements in reach performance (decreased endpoint error and movement time). The Left Arm group demonstrated an increased use of anticipatory planning and feedback-based adjustments to control reach extent with practice while the Right Arm group did not show a change. Changes in the control of reach extent seen in the Left Arm group may reflect experience-dependent creation of a more robust internal model of the arm and/or increased weighting of control mechanisms from the dominant left-brain hemisphere. The potential to modify the use of anticipatory planning and feedback-based adjustments to control reach movements may be relevant for rehabilitation approaches in clinical populations such as stroke who have altered control of reach extent.

**Supplementary Information:**

The online version contains supplementary material available at 10.1007/s00221-025-07181-x.

## Introduction

The central nervous system uses a combination of anticipatory planning and feedback-based adjustments to successfully execute a goal-directed reaching movement (Desmurget and Grafton 2000). Previous experience with the demands of the task (e.g., initial target and hand locations) and knowledge of the properties of the arm are used to formulate an initial motor plan prior to movement onset (Desmurget and Grafton 2000; Sabes 2000). Upon movement onset, an efference copy is used to predict the sensory consequences of the movement and to actively compare the actual arm movement to predicted arm movement. Any discrepancies trigger feedback-based adjustments to support achievement of the desired goal (Desmurget and Grafton 2000; Sabes 2000).

A rapid goal-directed multi-joint reaching movement generally produces a velocity profile consisting of an acceleration phase followed by an inversely related deceleration phase. The magnitude and duration of these phases systematically scale for reaches to targets that vary in distance (Gordon et al. 1994b; Messier and Kalaska 1999; Sainburg and Schaefer 2004; Schaefer and Sainburg 2008; Stewart et al. 2013). Features of the acceleration phase that occur prior to peak velocity can be used to infer the use of anticipatory planning and early feedback-based adjustments to control the actual distance moved (Sainburg and Schaefer 2004; Stewart et al. 2013, 2014b). When reaching to targets that vary in distance, scaling of the magnitude of the initial peak of acceleration, which occurs shortly after movement onset before sensory feedback is available to make corrections, is indicative of the use of anticipatory planning to control movement distance (Brown and Cook 1981; Gordon and Ghez 1987a; Sainburg and Schaefer 2004; Stewart et al. 2013). Scaling of acceleration duration is indicative of early feedback-based adjustments to the reach trajectory to compensate for the errors in the initial motor plan (Brown and Cooke 1984; Gordon and Ghez 1987b; Mutha and Sainburg 2007; Sainburg and Schaefer 2004; Stewart et al. 2013). This systematic scaling of acceleration magnitude and duration and their ability to predict the actual distance moved suggests that the central nervous system has knowledge (i.e. an internal model) of the arm’s kinematics and dynamics (Desmurget and Grafton 2000; Sabes 2000).

It was first observed over a century ago that repeated practice of a movement results in improved motor performance (Woodworth 1899). Since then, it has been shown that even simple reaching movements show improvements in performance with repetitive practice (Gottlieb et al. 1988). Notably, practice can lead to changes in movement velocity and acceleration, end-point accuracy, and features of the acceleration/deceleration profile (Corcos et al. 1993; Flament et al. 1999; Gottlieb et al. 1988). However, it is not known if repetitive practice leads to changes in the use of anticipatory planning and feedback-based adjustments for the control of reach extent during three-dimensional (3D), multi-joint reaching movements.

The dominant right arm and the non-dominant left arm may respond differently to a period of practice. During two-dimensional (2D) planar reaches, the dominant right arm demonstrates greater reliance on anticipatory planning (scaling of acceleration magnitude) while the non-dominant left arm demonstrates greater reliance on feedback-based adjustments (scaling of acceleration duration) to control reach extent (Sainburg and Schaefer 2004; Schaefer et al. 2007). However, these patterns may or may not be present when reaching movements are performed in a less constrained 3D environment (Schaffer and Sainburg 2017; Stewart et al. 2013) and the two arms may respond differently to a period of practice. Additionally, during everyday life, the nondominant arm is used less than the dominant arm (Bailey and Lang 2013; Kilbreath and Heard 2005; Vega-González and Granat 2005), which may lead to a less robust internal model of the arm. As such, knowledge of the non-dominant left arm gained through repetitive practice of reaching may lead to an updated internal model of the arm that results in greater increases in the use of anticipatory planning and feedback-based adjustments than the dominant right arm.

The purpose of this study was to examine the effects of three days of practice on the control of reach extent for reaches in 3D space. We hypothesized that both arms would exhibit improved reach performance (decreased movement time and endpoint error) with practice. We also hypothesized that the non-dominant left arm would show greater changes in the use of anticipatory planning (scaling of acceleration magnitude to movement distance) and feedback-based adjustments (scaling of acceleration duration to movement distance) to control react extent with repetitive practice than the dominant right arm as individuals gained knowledge about their arm.

## Methods

### Participants

Twenty-four, right-hand dominant, non-disabled adults completed the 3D reaching task for three consecutive days: 12 with the dominant right arm (5 female/7 male, mean age 22.5 ± 3.3 years) and 12 with the non-dominant left arm (8 female/4 male, mean age 24.6 ± 1.1 years). All participants were right-hand dominant as determined by the Edinburgh Handedness Questionnaire (Oldfield 1971). No participant had a present or prior history of neurological disorders or current pain in the arm. A power analysis determined that this sample size would provide 80% power to find a medium effect with a mixed model analysis of variance (f = 0.27, α = 0.05, 2 groups, 3 measures; G.Power 3.1.9.7). All participants provided written consent prior to participation in the study according to a protocol approved by the University of South Carolina’s Institutional Review Board.

### Reaching task

**Fig. 1:**
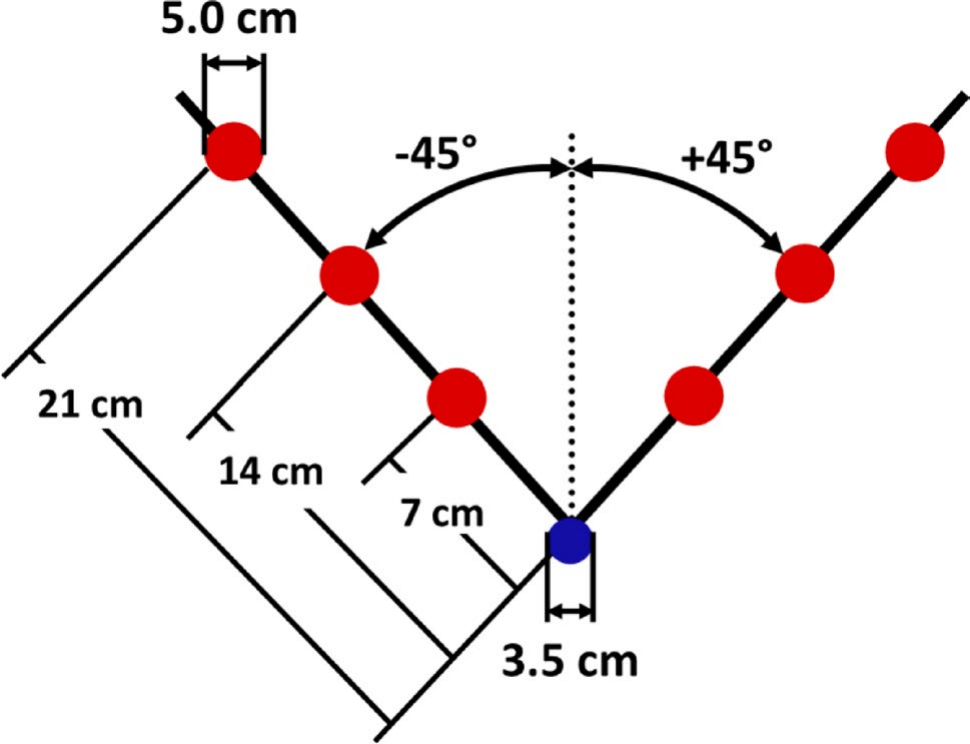
Experimental setup. Goal targets (5.0 cm red sphere) were presented one at a time in a virtual workspace in two directions (45° to the right and left of midline) and 3 distances (7, 14, 21 cm). All reach trials started at a Start target (3.5 cm blue sphere) at participant midline

Participants reached to six spherical targets (5 cm diameter) presented in two directions (45° to the right and 45° to the left of midline) and three distances (7, 14, 21 cm) in a virtual environment (Innovative Sports Training, Inc., Chicago, IL) (Fig. 1). Participants wore stereoscopic glasses sampled at 60 Hz per eye to provide 3D visualization of colored spheres against a black background. An electromagnetic sensor on the index finger of the tested arm represented finger position in the virtual environment (white sphere, 3.0 cm diameter); this same sensor captured position data throughout the reach (sampled at 120 Hz). Participants were instructed to “reach to the target as fast as possible when ready”; reach speed was emphasized over reach accuracy. Participants reached with the dominant right arm or the non-dominant left arm according to their assigned group (Right Arm, Left Arm). For analysis, target direction was defined as ipsilateral (right arm reaching to + 45° targets, left arm reaching to -45° targets) and contralateral (right arm reaching to −45° targets, left arm reaching to + 45° targets) to allow direct comparison between arms for reaches with similar biomechanical demands.

At the start of each trial, participants moved a Cursor that represented finger position to a Start target at midline (blue sphere, 3.5 cm diameter, error tolerance of 1.0 cm). After maintaining the Cursor in the Start position for 1 s, the Start target turned yellow as a ready signal. Following a variable period of 1.3, 1.6–1.9 s, the Start target and Cursor disappeared, and a single Goal target appeared (red sphere, 5.0 cm diameter). Accordingly, online visual feedback of hand position (Cursor) was not available during the reach. After each reach was complete, visual feedback was provided showing the Goal target and final Cursor position. If the Cursor overlapped with the Goal target (error tolerance of 4.0 cm), the Goal target turned green during post-trial feedback. If the Cursor did not overlap with the Goal target, the Goal target remained red during post-trial feedback.

### Experimental procedure

On Day 1, participants were first provided exposure to the virtual environment to orient to the workspace and target locations. During this exposure trial, all six Goal targets, the Start target, and the Cursor representing index finger position were visible. Next, one practice block of 10 reach trials was performed to ensure understanding of the trial sequence. Participants then completed 8 blocks of 24 trials per block (192 total trials). Goal targets were presented in a pseudorandom order such that each block included 4 trials to each of the six Goal targets and no consecutive trials were to the same target. Day 2 and Day 3 included 8 blocks of 24 reach trials (192 total trials) similar to Day 1.

### Data analysis

Data were analyzed offline in MATLAB using custom scripts (MathWorks, Natick, MA). Kinematic data collected from the electromagnetic sensor were low-pass filtered (2nd order Butterworth, 10 Hz cut-off) and differentiated to obtain velocity and acceleration (Winter 2005). Movement onset was determined by searching backward in time from the time of peak velocity until velocity dropped below 10 cm/s and either increased again or the change in velocity was low (< 3 cm/s). Movement offset was determined by searching forward in time from the time of peak velocity until velocity dropped below a minimum value (20 cm/s if peak velocity > 60 cm/s, 10 cm/s if peak velocity < 60 cm/s) and either velocity changed direction (increased again) or the change in velocity was very low (< 1 cm/s). Each trial was visually inspected to ensure accuracy in the determination of movement onset and offset.

The primary reach performance variables were movement distance, movement time, and endpoint error. Movement distance was the 3D linear distance (centimeters) between the position of the hand at movement onset and movement offset. Movement time was the time (seconds) between movement onset and movement offset. Endpoint error was the 3D linear distance (centimeters) between the final position of the index finger at movement offset and the center of the target. Secondary measures of reach performance included peak velocity, time of peak velocity, and peak acceleration. Peak velocity (cm/s) was found by searching forward in time to the first peak of velocity after movement onset. Time of peak velocity was the time (seconds) corresponding to the first velocity peak. Peak acceleration (cm/s^2^) was found by searching forward for the first acceleration peak between movement onset and the time of peak velocity.

**Fig. 2:**
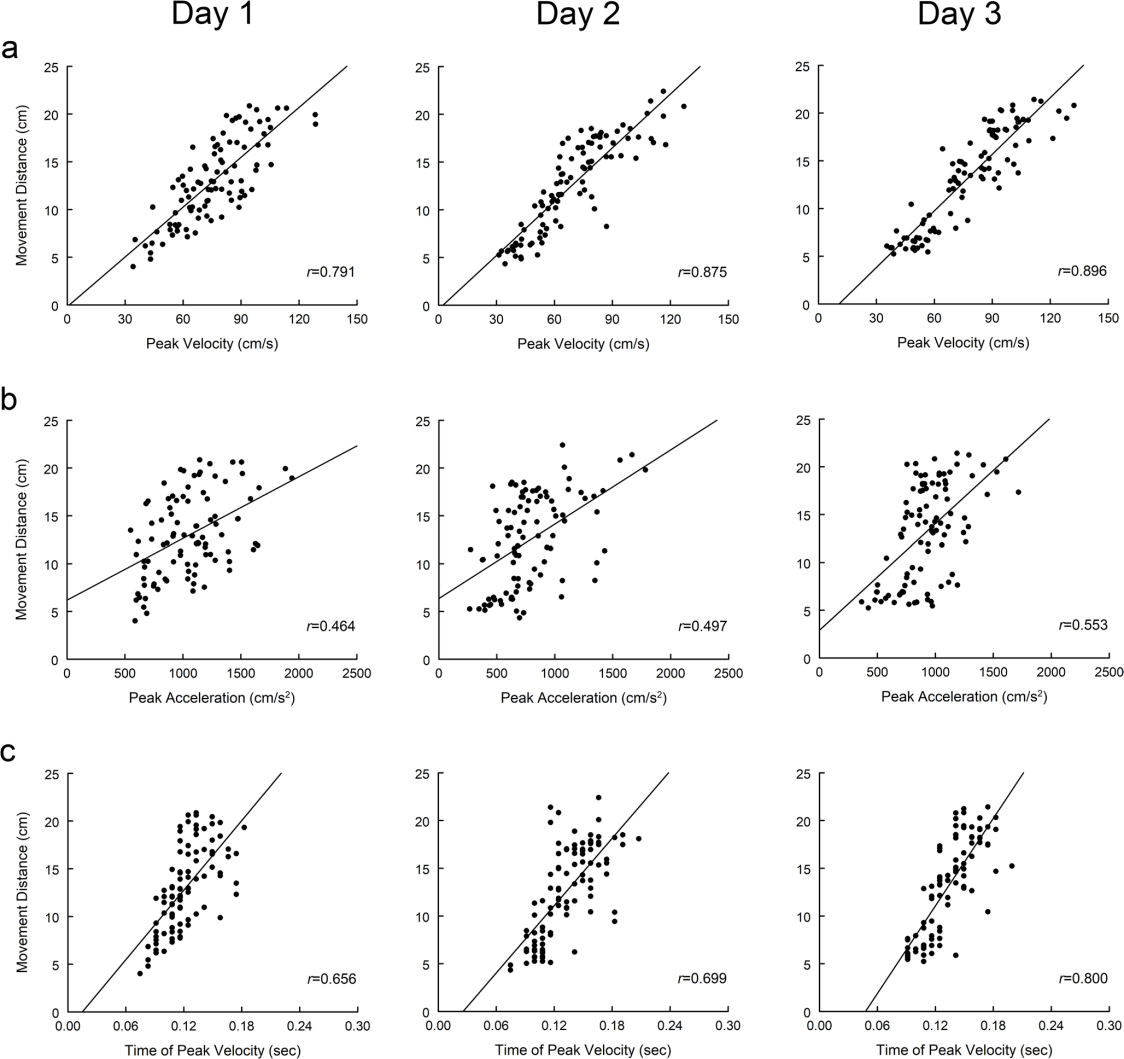
Relationship between early kinematic variables to movement distance for a single participant reaching with the left arm to the contralateral targets over three days of practice (Day 1, Day 2, Day 3). Scatter plots show how peak velocity (**a**), peak acceleration (**b**), and time to peak velocity (**c**) predict actual movement distance across all targets (each circle represents a single reach trial). Linear regression line fit to the data represented by the solid line. *r* is the correlation coefficient between the kinematic variables and movement distance

The utilization of anticipatory planning and feedback-based adjustments was determined for each individual participant by examining how well early kinematic variables (peak acceleration, time of peak velocity) predicted the actual distance moved (Sainburg and Schaefer 2004; Stewart et al. 2013). Analyses were completed separately for each direction (ipsilateral, contralateral) and for each day of practice. Anticipatory planning was defined as the correlation between peak acceleration and movement distance (*r*_PA_); a significant, positive correlation demonstrates a scaling of peak acceleration relative to movement distance and the use of planning to control reach extent (Fig. 2b). Feedback-based adjustments were defined as the correlation between time of peak velocity and movement distance (*r*_TPV_); a significant, positive correlation demonstrates a scaling of the time of peak velocity (or acceleration duration) relative to movement distance and the use of feedback-based adjustment prior to peak velocity to control reach extent (Fig. 2c). The overall initial control pattern was defined by how well peak velocity correlated with movement distance (*r*_PV_, Fig. 2a). The strength of the correlations was assessed based on the value of *r*: *r* < 0.25 = little to none; 0.25 < *r* < 0.50 = fair, 0.50 < *r* < 0.75 = moderate; *r* > 0.75 = strong (Portney and Watkins 2009). The *r* values were used to report the results, however, correlation coefficients were transformed to a Fisher Z score for statistical analysis.

### Statistical analysis

Data from Day 1 were used to define baseline performance. Reach performance on Day 1 was examined for primary (movement distance, movement time, endpoint error) and secondary measures (peak velocity, time of peak velocity, and peak acceleration) with a mixed model analysis of variance (ANOVA) with within group factors for target direction (ipsilateral, contralateral) and target distance (7 cm, 14 cm, 21 cm) and a between group factor for arm group (Right Arm, Left Arm). Baseline control of reach extent (anticipatory planning, feedback-based adjustments) was examined with a mixed model analysis of variance (ANOVA) that included a within group factor for target direction and a between group factor for arm group.

Data from all three days were used to examine the effects of practice on reach performance and the control of reach extent. Mixed model ANOVAs that included within group factors of target distance and days (Day 1, Day 2, Day 3) and a between group factor for arm group were used to examine the effects of practice on reach performance. To simplify interpretation, analyses of reach performance variables were completed separately for the ipsilateral and contralateral directions given the well described effect of reach direction on reach performance measures (Gordon et al. 1994a, b; Stewart et al. 2013). Mixed model ANOVAs that included a within group factor for days and a between group factor for arm group were used to examine the effects of practice on the control of reach extent (*r*_PA_, *r*_TPV_, *r*_PV_). When a significant interaction was found that included the arm group or target direction factor, a repeated measures ANOVA was completed separately for each arm group or target direction. Partial eta squared (η^2^) was used to estimate effect size where η^2^ = 0.01–0.059 was considered a small effect, η^2^ = 0.06–0.139 was considered a medium effect, and η^2^ *≥* 0.14 was considered a large effect (Cohen 1988). For all statistical tests, the significance was set at *p* < 0.05.

## Results

### Baseline reach performance and control of reach extent

**Fig. 3:**
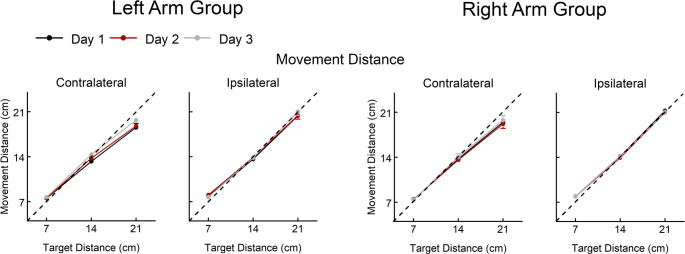
Movement distance for reaches in the contralateral and ipsilateral directions for each day of practice (Day 1, Day 2, Day 3) for the Left Arm Group and the Right Arm Group. Data presented as mean ± standard error. Dotted line indicates perfect scaling of movement distance to target distance

**Fig. 4:**
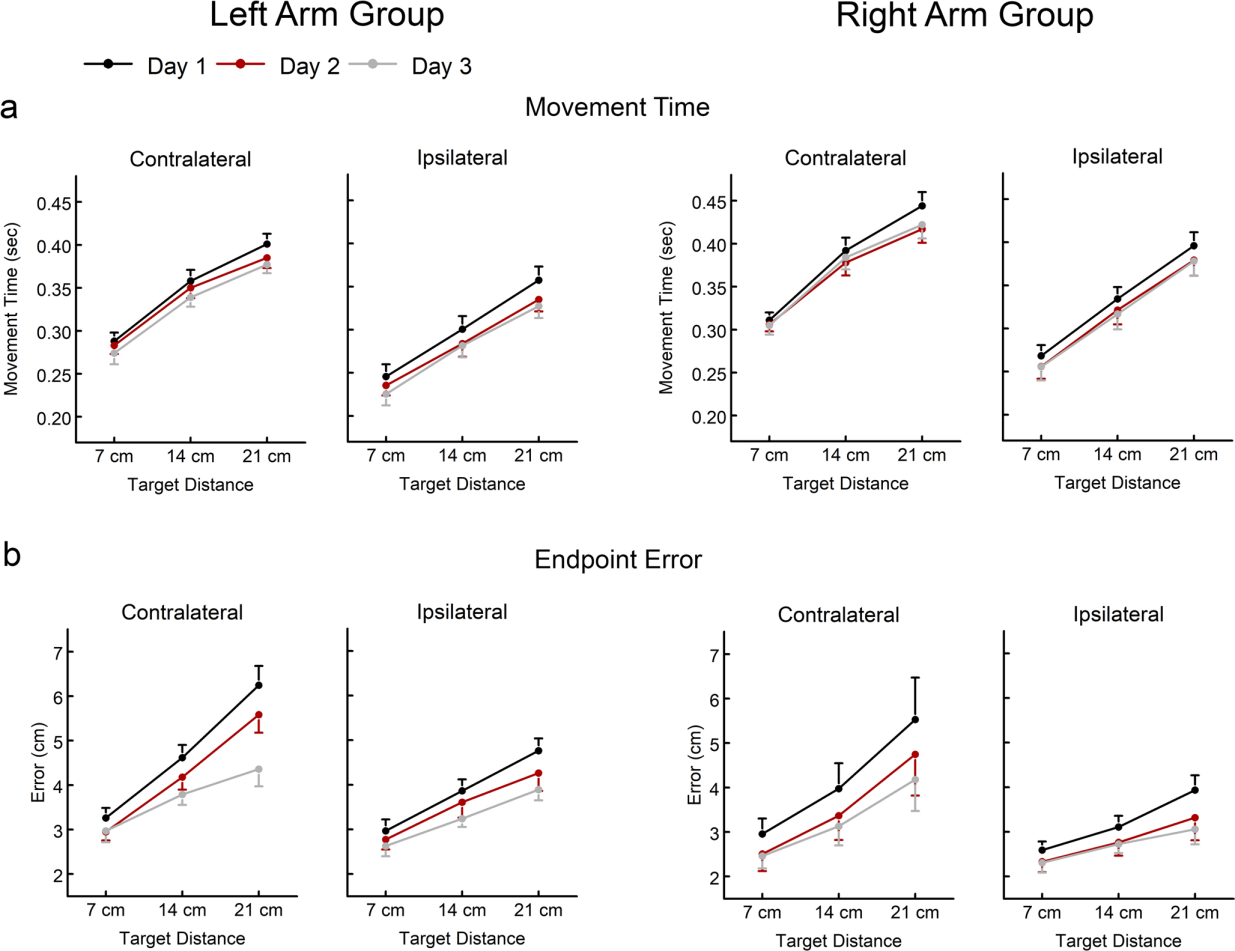
Movement time (**a**) and endpoint error (**b**) for reaches in the contralateral and ipsilateral directions for each day of practice (Day 1, Day 2, Day 3) for the Left Arm Group and the Right Arm Group. Data presented as mean ± standard error

Day 1 performance was used to examine baseline reach performance (Figs. 3 and 4). As expected, all three primary measures of reach performance systematically scaled to target distance (movement distance: F = 726.993, *p* < 0.001, η^2^ = 0.986; movement time: F = 216.855, *p* < 0.001, η^2^ = 0.954; endpoint error: F = 32.257, *p* = < 0.001, η^2^ = 0.754). Reaches in the contralateral direction tended to have shorter movement distances that undershot the target (F = 12.319, *p* = 0.002, η^2^ = 0.359), longer movement times (F = 86.170, *p* < 0.001; η^2^ = 0.797) and larger endpoint errors (F = 15.426, *p* < 0.001, η^2^ = 0.412) than reaches in the ipsilateral direction. While the Right Arm and Left Arm groups had similar movement distances (F = 1.113, *p* = 0.303, η^2^ = 0.048), the Left Arm group had slightly shorter movement times and slightly larger endpoint errors, though these differences were not significant (movement time: F = 3.435, *p* = 0.077, η^2^ = 0.135; endpoint error: (F = 1.621, *p* = 0.216, η^2^ = 0.069). Baseline analyses of secondary measures of reach performance are reported in the Supplemental Results.

**Fig. 5:**
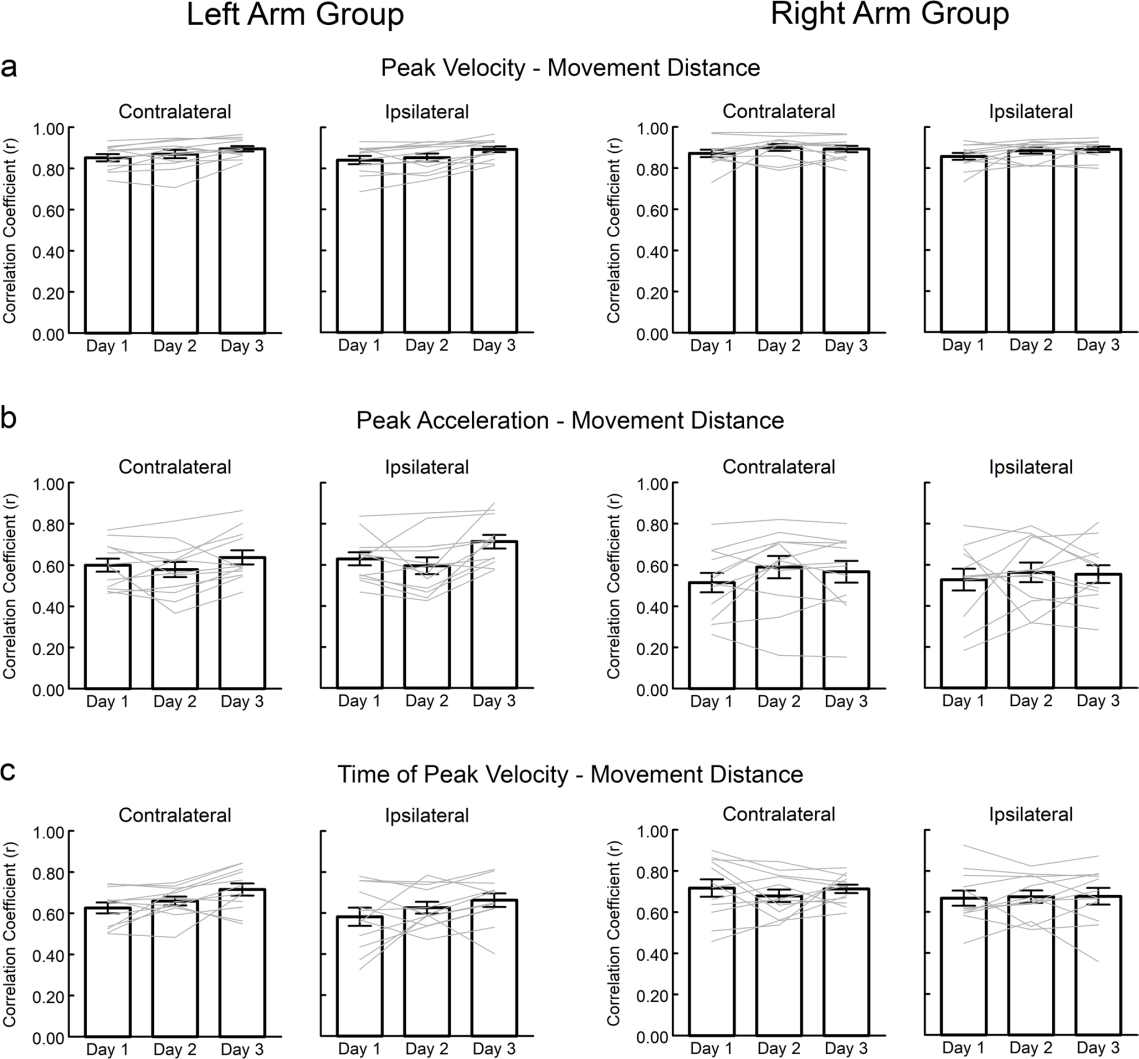
Correlation coefficients (*r*) between kinematic variables and movement distance for the Left Arm Group and Right Arm Group in the contralateral and ipsilateral directions over three days of practice: **a** Peak velocity with movement distance (*r*_PV_) is representative of the overall early control pattern; **b** Peak acceleration with movement distance (*r*_PA_) is representative of anticipatory planning; **c** Time of peak velocity with movement distance (*r*_TPV_) is representative of feedback-based adjustments. Bars represent the group mean ± standard error; grey lines represent data for each individual participant

Day 1 performance was also used to examine baseline control of reach extent. Correlations between early reach kinematics and final movement distance are illustrated for a single participant in the Left Arm group (Fig. 2) and for all participants in each group (Fig. 5). Peak velocity was a strong and significant predictor of movement distance across participants for both the ipsilateral (mean *r*_PV_ = 0.849 ± 0.064) and contralateral (mean *r*_PV_ = 0.862 ± 0.061) directions consistent with previous cross-sectional studies (Messier and Kalaska 1999; Sainburg and Schaefer 2004; Stewart et al. 2013); higher peaks of velocity corresponded to longer movement distances while lower peaks of velocity corresponded to shorter movement distances. The correlation between peak velocity and movement distance did not differ between target directions (F = 0.830, *p* = 0.372, η^2^ = 0.036) or arm groups (F = 2.624, *p* = 0.120, η^2^ = 0.107). Peak acceleration and time of peak velocity were moderate predictors of movement distance (mean *r*_PA_: ipsilateral 0.579 ± 0.157, contralateral 0.558 ± 0.143; mean *r*_TPV_: ipsilateral 0.625 ± 0.145, contralateral 0.671 ± 0.128). These findings suggest participants utilized a combination of anticipatory planning and feedback-based adjustments to control reach extent (Stewart et al. 2013). Correlations between peak acceleration and movement distance (*r*_PA_) did not differ between the ipsilateral and contralateral directions (F = 1.602, *p* = 0.219, η^2^ = 0.068) or between the Right Arm and Left Arm groups (F = 0.827, *p* = 0.373, η^2^ = 0.036). In addition, correlations between time of peak velocity and movement distance (*r*_TPV_) did not differ between the ipsilateral and contralateral directions (F = 1.984, *p* = 0.173, η^2^ = 0.083), however, they were higher in the Right Arm group compared to the Left Arm group (F = 4.432, *p* = 0.047, η^2^ = 0.168).

### Effect of practice on reach performance

Over three days of practice, kinematic variables of reach performance continued to systematically scale to target distance and differ based on target direction (Figs. 3 and 4). Movement distance did not change with practice for reaches in the ipsilateral direction (F = 0.078, *p* = 0.925, η^2^ = 0.007) but did change for reaches in the contralateral direction that varied with target distance (day x distance interaction: F = 9.080, *p* < 0.001, η^2^ = 0.657). Post-hoc analyses of the contralateral reaches run separately for each distance found that movement distance increased slightly over practice for reaches for the 14 cm target distance (F = 4.539, *p* = 0.022, η^2^ = 0.292, mean change = 0.70 cm) and the 21 cm target distance (F = 10.186, *p* < 0.001, η^2^ = 0.481, mean change = 0.72 cm) but not the 7 cm target distance (F = 0.030, p =. 0.970, η^2^ = 0.003, mean change = 0.06 cm).

Overall, movement time and endpoint error improved with practice. Movement time decreased with practice for both the ipsilateral direction (F = 4.400, *p* = 0.025, η^2^ = 0.295, mean change = 0.020 s) and the contralateral direction (F = 3.772, *p* = 0.04, η^2^ = 0.264, mean change = 0.015 s). The Left Arm group continued to have shorter movement times than the Right Arm group for reaches in the contralateral direction (F = 4.946, *p* = 0.037, η^2^ = 0.184) but not for reaches in the ipsilateral direction (F = 3.144, *p* = 0.09, η^2^ = 0.125). Endpoint error also decreased with practice for both the ipsilateral direction (F = 6.436, *p* = 0.007, η^2^ = 0.380, mean change = 0.56 cm) and the contralateral direction (F = 10.185, *p* < 0.001, η^2^ = 0.492, mean change = 0.95 cm). While endpoint error was slightly higher for the Left Arm group compared to the Right Arm group, these differences were not statistically significant (ipsilateral: F = 4.239, *p* = 0.052, η^2^ = 0.162; contralateral: F = 0.989, *p* = 0.331, η^2^ = 0.043). Analyses of secondary measures of reach performance are reported in the Supplemental Results.

### Effect of practice on the control of reach extent

The correlation between peak velocity and movement distance, representative of the overall early control of reach extent, changed with practice (Fig. 5a). Practice effects differed between the ipsilateral and contralateral directions (days × direction interaction: F = 10.114, *p* < 0.001, η^2^ = 0.491), however, there was no difference between arm groups (F = 0.637, *p* = 0.434, η^2^ = 0.028). Post-hoc analyses found that the correlations increased with practice in both directions but that the increase was greater for the ipsilateral direction (mean *r*_*PV*_ change = 0.044) than for the contralateral direction (mean *r*_*PV*_ change = 0.032). The correlation between peak acceleration and movement distance, indicative of anticipatory planning to control reach extent, also changed over three days of practice (Fig. 5b), however, the change differed between arm groups (days × group interaction: F = 4.073, *p* = 0.032, η^2^ = 0.280). Post-hoc analyses found that the correlation increased in the Left Arm group (F = 4.417, *p* = 0.042, η^2^ = 0.469, mean *r*_PA_ change: = 0.06) but not in the Right Arm group (F = 1.015, *p* = 0.397, η^2^ = 0.169, mean *r*_PA_ change = 0.04). Pairwise comparisons on each day found no significant differences between the two arms groups in *r*_PA_ (*p* > 0.06 after Bonferroni correction). Finally, the correlation between time to peak velocity and movement distance, indicative of early feedback-based adjustments to control reach extent, also changed over three days of practice (Fig. 5c), however, this change differed between arm groups (days × group interaction: F = 4.041, *p* = 0.033, η^2^ = 0.278). Post-hoc analyses found that the correlation increased with practice in the Left Arm group (F = 8.483, *p* = 0.007, η^2^ = 0.629, mean *r*_TPV_ change = 0.085) but not the Right Arm group (F = 0.823, *p* = 0.467, η^2^ = 0.141, mean *r*_TPV_ change = 0.002). Pairwise comparisons on each day found that *r*_TPV_ was higher in the Right Arm group than the Left Arm group on Day 1 (*p* = 0.047) but did not differ between the two groups on Day 2 or 3 (*p* > 0.224 after Bonferroni correction).

## Discussion

The present study investigated the effect of three days of practice on the control of reach extent. At baseline, participants utilized a combination of anticipatory planning (*r*_PA_) and early feedback-based adjustments (*r*_TPV_) to control reach extent. Over three days of practice, the Left Arm group demonstrated increased use of anticipatory planning and feedback-based adjustments to control reach extent, although the magnitude of the changes were relatively small. In contrast, the Right Arm group did not show significant changes to the control of reach extent. As expected, both arm groups showed practice-related changes in reach performance consistent with previous studies demonstrating improvements with the practice of simple reaching tasks (Gottlieb et al. 1988). Overall, the results of this study suggest that the control of reach extent may be modifiable with practice.

### Effect of practice on the control of reach extent

At baseline, both arm groups used a combination of anticipatory planning and feedback-based adjustments to control reach extent in both target directions. This is similar to a previous study that examined right arm reaches using the same 3D paradigm (Stewart et al. 2013) but differs from another study that examined the control of reach extent in 2D, planar reaches (Sainburg and Schaefer 2004). In that study, reaches with the non-dominant left arm relied more on feedback-based adjustments than anticipatory planning whereas reaches with the dominant right arm relied more on anticipatory planning than on feedback-based adjustments. In the current study, the control of reach extent may have been influenced by the task environment. Differences in reach control between the nondominant and dominant arms in more constrained environments may not fully translate to reaches in 3D (Schaffer and Sainburg 2017).

After 3 days of practice, the control of reach extent changed in the Left arm group but not the Right arm group. The Left Arm group demonstrated statistically significant increases with large effect sizes in the use of both anticipatory planning (*r*_PA_) and early feedback-based adjustments (*r*_TPV_) to control reach extent. Overall, this finding indicates that the magnitude of peak acceleration and the duration of the acceleration phase (i.e. time of peak velocity) became a stronger and more consistent predictor of the actual distance moved over the three days of practice. However, the magnitude of these changes with practice in the Left Arm group were relatively small (mean *r* change of 0.06 and 0.085). The small magnitude of the changes may have been due to the moderate to strong correlations found at baseline limiting the available range for improvement. Nevertheless, changes in the control of reach extent were found in the Left Arm group reaching with the non-dominant arm that were not observed in the Right Arm group reaching with the dominant arm.

Our results suggest that repetitive reach practice with the non-dominant, left arm facilitated increased knowledge about the arm which was used to increase the use of anticipatory planning and early feedback-based adjustments for the control of reach extent. In contrast, individuals reaching with the dominant, right arm did not show a change in the control of reach extent with practice. The dominant arm is used more than the nondominant arm for everyday goal-directed tasks (Bailey and Lang 2013; Kilbreath and Heard 2005; Vega-González and Granat 2005). Greater use may create a more robust internal model of the arm. A bout of repetitive practice of reach movements with the non-dominant arm may provide sensorimotor experience that increases the central nervous system’s knowledge of the properties of the arm, knowledge that is used to improve the internal model of the arm.

The lack of change in the control of reach extent with practice in the Right Arm group may have been due to between-participant variability and lack of consistency over days, especially for anticipatory planning (Fig. 5b). On Day 1, *r*_PA_ for the Right Arm group ranged from 0.183 to 0.796 compared to 0.464 to 0.837 for the Left Arm group with the variability persisting over days. This range for the Right Arm group was larger than the range reported in a previous study using the same paradigm, although the sample size in that study was smaller (*n* = 6) and the task was only completed for one day (Stewart et al. 2013). The reason behind this variability with the right arm is not fully clear. Overall, each individual reaching with dominant, right arm appeared to select a control pattern that utilized planning and feedback-based adjustments to whatever degree they desired in order to produce the intended reach. Multiple solutions may have been available to individuals reaching with the dominant arm based on their previous movement experience (Ranganathan et al. 2014). The potential solution options may have been more limited for the nondominant arm leading to a more consistent pattern between participants. Based on the findings of the current study, future studies could be designed to provide a better understanding of this variability within and over days.

The complementary dominance hypothesis proposed by Kitchen et al. (2024) may also explain the changes in the control of reach extent with practice in the Left Arm group. That hypothesis proposes that both brain hemispheres contribute to the control of reaching movements in both arms with the hemisphere contralateral to the reaching arm having greater influence. The dominant left hemisphere of right-hand dominant individuals is thought to be specialized for optimal control of limb dynamics while the nondominant right hemisphere is thought to be specialized for impedance control under unpredictable conditions (Kitchen et al. 2024). Accordingly, the increased use of anticipatory planning and early feedback-based adjustments in the non-dominant left arm with practice in the present study may be due to increased contributions from the dominant hemisphere. Specifically, the Left arm group may have responded to practice of 3D reaching movements in a predictable task environment by increasing the contribution of the dominant left hemisphere (i.e. increased reliance on control of limb dynamics). This idea is supported by Philip and Frey (2014) that studied individuals with an amputation of their dominant right arm who learned to use the previously non-dominant left arm as the primary effector for upper extremity tasks. That study found that improvements in skilled motor performance in the left arm were associated with increased activation of the ipsilateral (dominant) left hemisphere, suggesting that the control of the left arm relied on increased contributions from the dominant left hemisphere.

### Effect of practice on reach performance

Consistent with previous literature, kinematic measures of reach performance scaled to target distance (Gordon et al. 1994a, b; Messier and Kalaska 1999; Sainburg and Schaefer 2004; Smith et al. 2022; Stewart et al. 2013) and differed based on target direction (Dounskaia 2005; Gordon et al. 1994a, b, 1995; Gritsenko et al. 2011). Overall, these patterns were maintained over the three days of practice as overall reach performance improved. Both arm groups exhibited decreases in movement time and endpoint error with practice, similar to previous work on the effect of practice on the performance of single-joint reaches (Gottlieb et al. 1988). These improvements in reach performance suggest that individuals increased their ability to meet the demands of the task - i.e. move the hand to target accurately and quickly. In contrast, the control of reach extent is indicative of how successful the initial motor command (peak acceleration magnitude) and early feedback-based adjustments (acceleration duration) are at achieving the eventual distance moved (Stewart et al. 2014a). In the current study, improvements in reach performance were achieved in combination with changes in the use of anticipatory planning and feedback-based adjustments for reaches with the nondominant left arm but not the dominant right arm suggesting that improvements in reach performance and changes in the control of reach extent are not inherently coupled.

### Clinical relevance

The results of this study may be relevant for individuals with altered control of reach extent after stroke. Stewart et al. (2014a, b) found that individuals with mild to moderate motor impairment due to stroke had a decreased use of anticipatory planning to control reach extent. After stroke, use of the more impaired arm is reduced (Bailey et al. 2015; Kim et al. 2022; Rand and Eng 2012; Waddell et al. 2019; Wolf et al. 2006) which may lead to reduced knowledge of the properties of the arm. The decreased use of anticipatory planning to control reach extent after stroke may represent this reduced knowledge of the arm. The results of the current study suggest that altered control of reach extent after stroke may be modifiable with practice and should be examined in future studies.

### Limitations

The present study had a few limitations. First, while the reaches were targeted, demands for endpoint accuracy were minimal which allowed participants to focus on moving quickly. It is possible the changes in the control of reach extent with practice may be different for a reaching task that requires endpoint accuracy (Smith et al. 2024). Second, the reaches were performed in a virtual environment which may impact how well these results translate to the real-world. However, a previous cross-sectional study comparing reaching in a virtual environment with reaching in a real-world environment showed that reaches with the dominant right arm utilized anticipatory planning and feedback-based adjustments similarly in both environments (Stewart et al. 2013). Third, the present study investigated the effect of three consecutive days of practice on the control of reach extent. Changes in the dominant right arm may have been observed with more days of practice. Last, our analysis focused on the effect of practice on early trajectory control. Feedback-based adjustments later in the movement (after the time of peak velocity) or control parameters focused on the final endpoint (Scheidt and Ghez 2007; Ghez et al. 2007) may have also changed with practice and could be considered in future studies.

## Conclusion

In conclusion, three days of practice led to changes in the control of reach extent in the Left Arm group but not the Right Arm group. The nondominant left arm demonstrated a small but significant increased use of anticipatory planning and feedback-based adjustments with practice that may be due to experience-dependent creation of a more robust internal model of the arm and/or to increased weighting of control mechanisms from the dominant left cerebral hemisphere. However, both arm groups demonstrated the expected improvements in overall reach performance (decreased movement time and endpoint error). The potential to modify the use of anticipatory planning and feedback-based adjustments to control reach extent may be relevant for rehabilitation approaches in clinical populations with altered control of reach extent.

## Supplementary Information

Below is the link to the electronic supplementary material.

Supplementary Material 1

## Data Availability

The datasets generated during and/or analyzed in the current study are available from the corresponding author on reasonable request.
