# Supplementary material for: Effect of practice on the control of reach extent
Source: Exp Brain Res. 2025 Oct 25;243(11):236. doi: 10.1007/s00221-025-07181-x (PMC12553570; doi:10.1007/s00221-025-07181-x)
Supplement: Supplementary file 1 — Supplementary Material 1 [file 221_2025_7181_MOESM1_ESM.pdf]

Supplemental Data

Effect of Practice on the Control of Reach Extent

Faith N Schroers[1], Troy M Herter[1], Dylan Bruemmer[1], Takeo Ichiyanagi[1],

Austin Hertherington[1], Michael O'Donnell[1], Janelle Ozorowski[1], Chad Simmons[1],

Jill Campbell Stewart[1§]

[1]*Department of Exercise Science, Arnold School of Public Health, University of South Carolina, Columbia SC*

§Corresponding Author: Jill Campbell Stewart, PT, PhD

University of South Carolina

921 Assembly Street, Room 301E

Columbia, SC 29208

Email: jcstewar@mailbox.sc.edu

Supplemental Results

Initial Reach Performance – Secondary Variables

Day 1 performance was used to examine baseline reach performance on secondary measures. As expected, peak velocity and peak acceleration scaled to target distance (peak velocity: F = 168.177, p <0.001, η^2 = 0.941; peak acceleration: F = 53.014, p <0.001, η^2 = 0.835) (Supplemental Figure). Additionally, both variables were higher in the ipsilateral direction than in the contralateral direction (peak velocity: F = 55.517, p <0.001, η^2 = 0.716; peak acceleration: F = 31.946, p <0.001, η^2 = 0.592). There was no difference between arm groups for peak velocity (F = 1.557, p = 0.225, η^2 = 0.066) or peak acceleration (F = 2.271, p = 0.146, η^2 = 0.094). Overall, time to peak velocity scaled to target distance (F = 47.469, p <0.001, η^2 = 0.819). Time to peak velocity occurred later for reaches in the contralateral direction compared to the ipsilateral direction (F = 7.297, p = 0.013, η^2 = 0.249) and for the Right Arm group compared to the Left Arm group (F = 3.957, p = 0.059, η^2 = 0.152), however, the latter comparison was not significant.

Effect of Practice on Secondary Reach Performance Variables

Peak velocity and peak acceleration increased with practice over days for reaches in the ipsilateral direction (peak velocity: F = 4.165, p = 0.030, η^2 = 0.284; peak acceleration: F = 4.821, p = 0.019, η^2 = 0.315) and for reaches in the contralateral direction (peak velocity: F = 5.804, p = 0.01, η^2 = 0.356; peak acceleration: F = 5.1, p = 0.016, η^2 = 0.327). For both variables, there was no significant difference between groups (p>0.094) and no significant group x day interaction (p>0.295). Time to peak

velocity decreased with practice for reaches in the ipsilateral direction ($F = 5.786$, $p = 0.010$, $\eta^2 = 0.355$). Similar to Day 1, time to peak velocity occurred earlier for the left arm group than for the right arm group for reaches in the ipsilateral direction ($F = 5.091$, $p = 0.034$, $\eta^2 = 0.188$). For reaches in the contralateral direction, time to peak velocity decreased with practice ($F = 4.657$, $p = 0.021$, $\eta^2 = 0.307$), however, the change over days differed between arm group (group x days interaction: $F = 3.686$, $p = 0.042$, $\eta^2 = 0.260$). Post-hoc analyses revealed the time to peak velocity significantly decreased in the Right Arm group ($F = 15.648$, $p < 0.001$, $\eta^2 = 0.758$) but not the Left Arm group ($F = 1.388$, $p= 0.294$, $\eta^2 = 0.217$) for reaches in the contralateral direction.

Supplement Figure

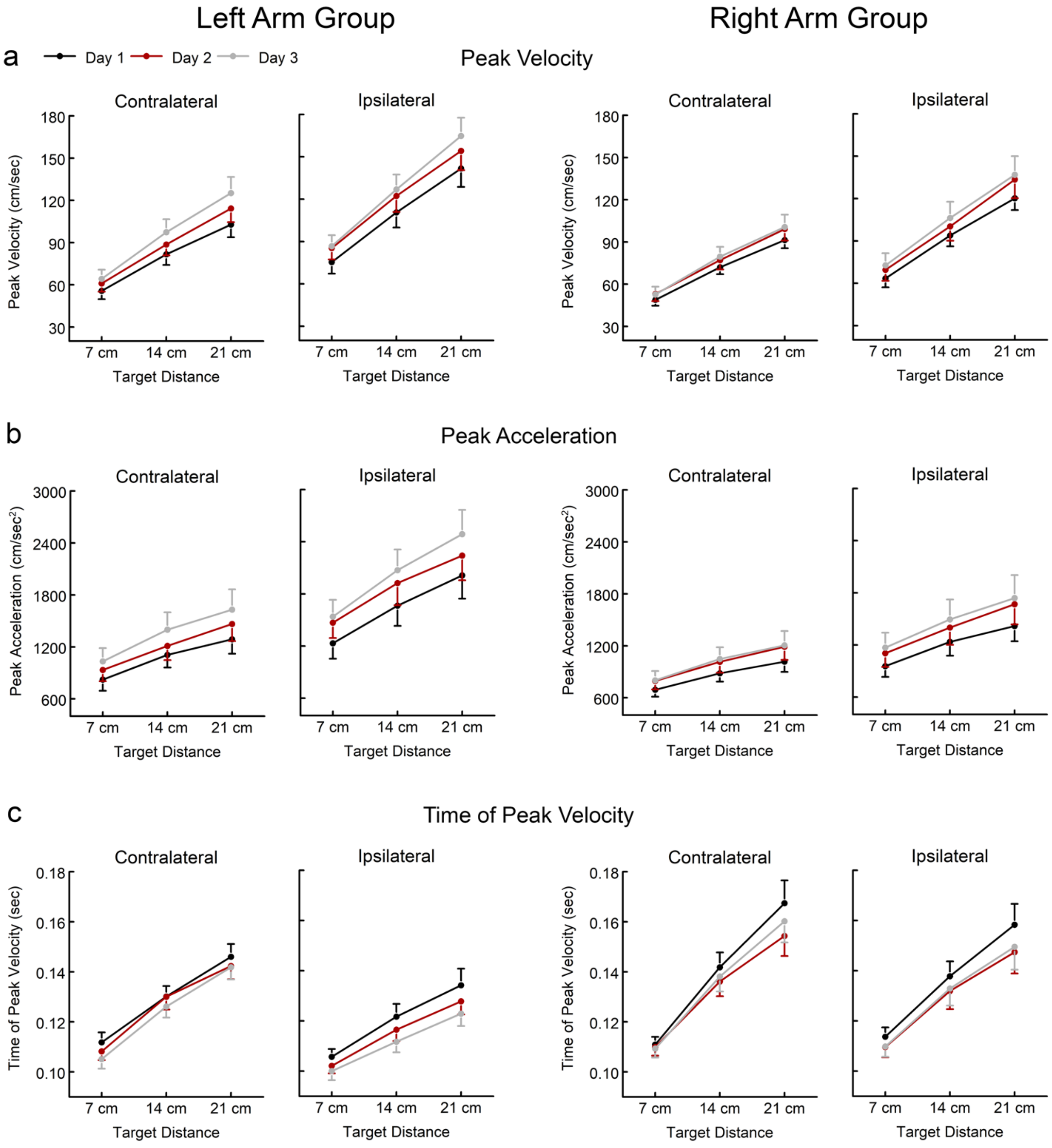

Supplemental Figure. Secondary measures of reach performance for reaches in the contralateral and ipsilateral directions over each day of practice (Day 1, Day 2, Day 3) for the Left Arm Group and the Right Arm Group: (a) peak velocity; (b) peak acceleration; (c) time to peak velocity. Data presented as mean ± standard error.
